# *In Vitro* Testing of Voltage Indicators: Archon1, ArcLightD, ASAP1, ASAP2s, ASAP3b, Bongwoori-Pos6, BeRST1, FlicR1, and Chi-VSFP-Butterfly

**DOI:** 10.1523/ENEURO.0060-20.2020

**Published:** 2020-09-04

**Authors:** Milena M. Milosevic, Jinyoung Jang, Eric J. McKimm, Mei Hong Zhu, Srdjan D. Antic

**Affiliations:** 1 Institute for Systems Genomics, Department of Neuroscience, UConn School of Medicine, Farmington, Connecticut 06030; 2 Center for Laser Microscopy, Faculty of Biology, University of Belgrade, Belgrade, Serbia

**Keywords:** ArcLight, Archon1, ASAP3b, Bongwoori, BeRST1, FlicR1, VSFP Butterfly

## Abstract

Genetically encoded voltage indicators (GEVIs) could potentially be used for mapping neural circuits at the plane of synaptic potentials and plateau potentials—two blind spots of GCaMP-based imaging. In the last year alone, several laboratories reported significant breakthroughs in the quality of GEVIs and the efficacy of the voltage imaging equipment. One major obstacle of using well performing GEVIs in the pursuit of interesting biological data is the process of transferring GEVIs between laboratories, as their reported qualities (e.g., membrane targeting, brightness, sensitivity, optical signal quality) are often difficult to reproduce outside of the laboratory of the GEVI origin. We have tested eight available GEVIs (Archon1, ArcLightD, ASAP1, ASAP2s, ASAP3b, Bongwoori-Pos6, FlicR1, and chi-VSFP-Butterfly) and two voltage-sensitive dyes (BeRST1 and di-4-ANEPPS). We used the same microscope, lens, and optical detector, while the light sources were interchanged. GEVI voltage imaging was attempted in the following three preparations: (1) cultured neurons, (2) HEK293 cells, and (3) mouse brain slices. Systematic measurements were successful only in HEK293 cells and brain slices. Despite the significant differences in brightness and dynamic response (ON rate), all tested indicators produced reasonable optical signals in brain slices and solid *in vitro* quality properties, in the range initially reported by the creator laboratories. Side-by-side comparisons between GEVIs and organic dyes obtained in HEK293 cells and brain slices by a “third party” (current data) will be useful for determining the right voltage indicator for a given research application.

## Visual Abstract

**Figure:**
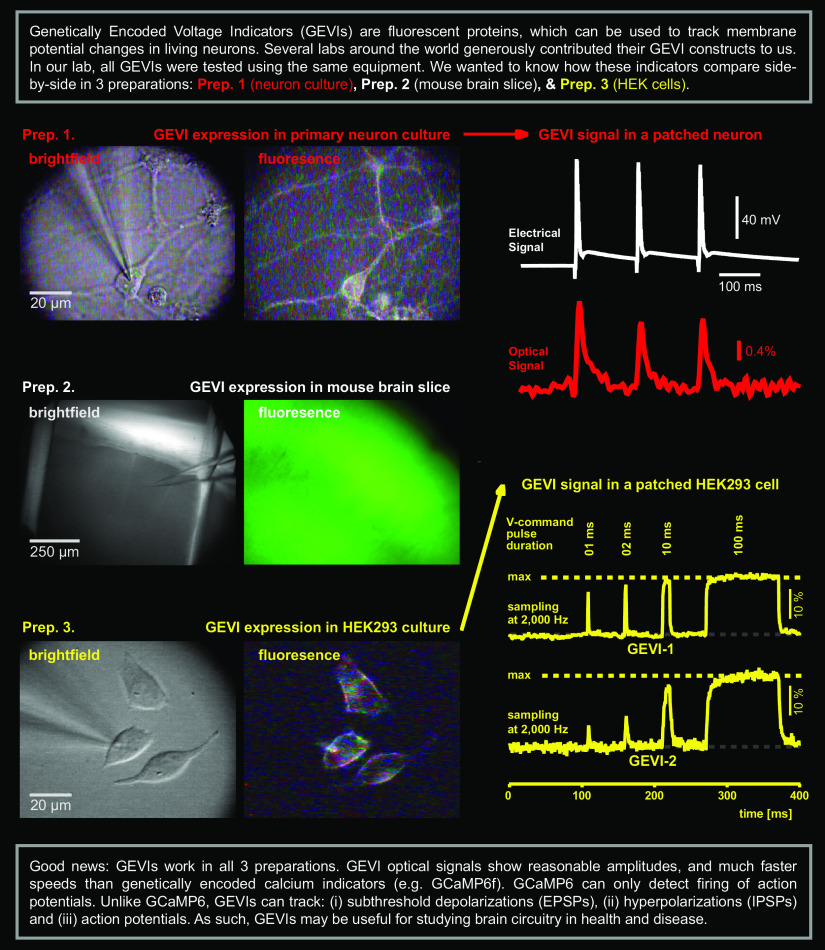

## Significance Statement

Voltage indicators are useful for studying brain circuitry and brain information processing, as they detect subthreshold neuronal signals missed by calcium indicators. But which voltage indicator should one use when planning a new (expensive) project? We performed systematic side-by-side testing of several popular genetically encoded voltage indicators (GEVIs), and then a voltage-sensitive dye was used in the same test. All reported measurements were acquired on the same electrophysiology-imaging station, using the same optical path and detector. Our results are potentially useful for guiding the practical choice of a GEVI indicator. We describe available excitation wavelengths, emission wavelengths, brightness, voltage sensitivity, and signal-to-noise ratio.

## Introduction

The exact cellular mechanisms by which mammalian brains experience sensations and generate decisions are largely unknown. Modern “brain research” efforts are directed toward mapping the connections between neurons and recording the activity of as many cells as possible (Lecoq et al., 2014; Chen et al., 2016; Tantirigama et al., 2017; Weisenburger et al., 2019). Optical imaging techniques, such as calcium imaging and voltage imaging, are particularly well suited for parallel recordings from multiple cells simultaneously. The choice between calcium and voltage should be based on the nature of the biological signal. When experimental designs require a simple detection of nerve impulses [also known as action potentials (APs)], calcium imaging is often superior to voltage imaging (Liang et al., 2018; Kerlin et al., 2019). When experimental designs require monitoring of subthreshold membrane potential changes, such as synaptic potentials and spikeless plateau depolarizations (Lampl et al., 1999; Volgushev et al., 2006), voltage imaging performs better than calcium imaging (Abdelfattah et al., 2019; Adam et al., 2019; Piatkevich et al., 2019; Villette et al., 2019). Therefore, comprehensive neuronal circuit analyses should be based on both GCaMP (calcium) and genetically encoded voltage indicator (GEVI; voltage) imaging data.

Although GEVI imaging shows some potential in neurobiology and systems neuroscience (Antic et al., 2016; Knöpfel and Song, 2019), it is not nearly as established and widely adopted as the GCaMP calcium imaging (Dana et al., 2014; Girven and Sparta, 2017). Simply, the GEVI optical signals were often too small to use in a real experiment (Bando et al., 2019; but see Storace et al., 2019). Systems neuroscientists are already overwhelmed with the complexity of their experiments (involving awake behaving animals and complex stimulation paradigms) to also worry about not getting any optical signals in GEVI imaging applications. Recently, we witnessed a sudden surge in the amount of research effort invested toward improving GEVI indicator properties and GEVI imaging equipment, mostly funded by the NIH Brain Research through Advancing Innovative Neurotechnologies (BRAIN) Initiative (Koroshetz et al., 2018). The latest improvements in the GEVI imaging field are quite impressive (Kannan et al., 2018; Abdelfattah et al., 2019; Adam et al., 2019; Piatkevich et al., 2019; Villette et al., 2019). However, sometimes GEVI variants do not perform as well as initially reported by their laboratory of origin. For example, in the original study, the GEVI probe “Ace2N” reported *in vivo* signals from the cell bodies and even dendrites of cortical pyramidal neurons (Gong et al., 2015), but the same construct, Ace2N, did not report any optical signals among all of the experimental conditions *in vivo* in an independent study (Bando et al., 2019). Another GEVI variant, “MacQ-mCitrine” (Gong et al., 2014) did not produce a stimulus-evoked response when averaging across all cells in the hands of a research team experienced with GEVI methodology (Chamberland et al., 2017). Although GEVI variants “ASAP1 and ASAP2s” are compatible with two-photon imaging (Yang et al., 2016) in imaging mouse visual cortex *in vivo* using two-photon excitation of ASAP1 or ASAP2s, Bando et al. (2019) did not detect reliable optical responses to visual stimulation (sampling intervals were too long perhaps). Clearly, the exciting GEVI performances reported by the laboratory of origin of the indicator have not been consistently replicated by the users in the field. Adoption of a GEVI imaging technology into an existing systems neuroscience research laboratory is a time-consuming, challenging, and risky process. Those who invested funding resources and personnel time, but failed to achieve adequate or reportable results, are fittingly reserved from the idea of using GEVIs in costly biological projects. Side-by-side comparisons of the existing GEVIs by an independent “third party” (Bando et al., 2019) could produce valuable practical data, potentially facilitating the use of GEVIs in meaningful experiments.

In the present project, we sought the most stable expression system that would allow us to compare the performances of several popular GEVIs. All of the recordings reported in the present article were obtained using the same microscope, objective lens, optical path, and CCD camera. When switching between two GEVIs of different excitation spectra, we toggled between light-emitting diode (LED; pE, CoolLed), metal halide lamp (Lumen 200, Prior Scientific), or semiconductor laser (OBIS, Coherent), attached to the same microscope port. We found that all GEVI indicators tested in the present study can produce quality population voltage imaging data (i.e., synaptically evoked compound synaptic potentials in the cortical neuropil). We found that ArcLight, Bongwoori, and VSFP express well, and are bright in brain neuropil. Consequently, these three indicators report good quality population (compound) signals. Furthermore, we found that Archon1 (red emission) and ASAP3b (green emission) are suitable for monitoring fast action potentials in individual cells, with ASAP being a slightly brighter and more forgiving probe. Our current data are potentially useful for guiding the practical choice of a GEVI indicator depending on the following: (1) biological application (e.g., cell body action potential; Abdelfattah et al., 2019); cell body UP state—a sustained ∼20 mV depolarization (Adam et al., 2019; Villette et al., 2019), dendritic back-propagating action potential (Gong et al., 2015; Adam et al., 2019), dendritic subthreshold depolarization (Kwon et al., 2017), compound excitatory synaptic potential (Storace and Cohen, 2017; Song et al., 2018), and compound inhibitory (hyperpolarizing) synaptic potential (Nakajima and Baker, 2018); (2) excitation wavelength; (3) emission wavelength; (4) brightness; (5) voltage sensitivity; and (6) signal-to-noise ratio.

## Materials and Methods

### Animals

Swiss Webster mice of either sex were used for the isolation of primary neurons [animal age, postnatal day 0.5 (P0.5)], as well as for the intracerebroventricular injections of genetically encoded voltage indicators packed in several variants of adeno-associated virus (AAV) backbones (animal ages, P0–P1), according to the animal protocols approved by the UConn Health Institutional Animal Care and Use Committee. For the evaluation of chimeric voltage-sensitive fluorescent protein (chi-VSFP), we used transgenic animals (C57BL/6 background) that express chi-VSFP (Song et al., 2017, 2018) in all cortical pyramidal neurons (CaMK2A-tTA; chi-VSFP), which were donated by Chenchen Song and Thomas Knöpfel (Imperial College London).

### GEVIs and dyes

FlicR1 (Abdelfattah et al., 2016) was provided by Ahmed Abdelfattah and Robert E. Campbell (University of Alberta, Edmonton, AB, Canada). ArcLightD (Jin et al., 2012) was provided by Jelena Platisa and Vincent Pieribone (Yale University, New Haven, CT). ASAP1 (St-Pierre et al., 2014) was provided by Mikhail Matlashov and Vlad Verkhusha (Albert Einstein College of Medicine, Bronx, NY). ASAP2s and ASAP3b (Chavarha et al., 2018) were provided by Guofeng Zhang and Michael Z. Lin (Stanford University, Stanford, CA). Bongwoori-Pos6 (Lee et al., 2017) was provided by Sungmoo Lee and Bradley J. Baker (Korea Institute of Science and Technology, Seoul, South Korea). BeRST1 (Huang et al., 2015) was provided by Evan Miller (University of California, Berkeley, Berkeley, CA). Archon1 (Piatkevich et al., 2018) was provided by Kiryl Piatkevich and Ed Boyden (MIT, Cambridge, MA). di-4-ANEPPS was purchased from Thermo Fisher Scientific (catalog #D1199).

### HEK293 cell culture and plasmid transfection

HEK293 cells were maintained in DMEM supplemented with 10% FBS, 2 mm GlutaMAX, 100 U/ml penicillin, and 100 μg/ml streptomycin. Cells were transfected in 24-well plates with 0.5 μg of DNA, using Lipofectamine 2000 (Thermo Fisher Scientific) according to the manufacturer instructions. One day after transfection, cells were seeded onto poly-l-ornithine-coated coverslips. GEVI voltage imaging was performed 1–2 d after seeding. Coverslips with transfected cells were washed in external solution and transferred to the microscope for imaging.

### HEK293 cell dye staining

For the experiments with voltage-sensitive dye, HEK293 cells were plated onto poly-l-ornithine-coated coverslips, and 1–2 d after seeding cells were treated with 1 μm BeRST1 in external solution for 15 min at 37°C. The external solution for voltage-sensitive dye staining contained the following (in mm): 125 NaCl, 0.5 KCl, 1 MgCl_2_, 3 CaCl_2_, 30 glucose, and 10 HEPES, pH 7.35 adjusted with NaOH (osmolality, ∼300 mOsm/kg). After dye treatment, cells were washed with external solution and transferred to the recording chamber on the microscope for imaging.

### Neuron culture and AAV transduction

Primary cortical and hippocampal neurons were isolated from newborn pups (P0.5) of Swiss Webster mice, according to the modified procedure by Beaudoin et al. (2012). Briefly, relevant brain structures were isolated in dissecting medium (DM) consisting of the following: Invitrogen HBSS without calcium and magnesium (catalog #14175095, Thermo Fisher Scientific) supplemented with sodium pyruvate 1 mm, glucose 0.02% (w/v), and HEPES 10 mm. The tissue was washed three times in DM, followed by enzymatic digestion at 37°C in the water bath in DM with trypsin (1 mg/ml for cortices and 0.5 mg/ml for hippocampi; freshly dissolved before this step; catalog #T4799, Sigma-Aldrich), for 13 min in the case of hippocampi or 20 min in the case of cortices, with occasional mixing. After the enzymatic digestion, DNase was added to the solution (0.1% final concentration) and incubated for an additional 5 min at room temperature. At this point, the tissue structure was loosened and settled on the bottom of the tube. The tissue was carefully washed three times in DM, an additional three times in temperature-equilibrated plating medium (PM) consisting of Invitrogen Minimum Essential Eagle’s Medium with Earle’s balanced salt solution (catalog #21010046, Thermo Fisher Scientific) supplemented with FBS 10%, glucose 0.09%, sodium pyruvate 1 mm, GlutaMAX 2 mm, penicillin 100 U/ml, and streptomycin 100 μg/ml. Finally, the loosened tissue was carefully mechanically digested in 1 ml of PM with a 1 ml pipette tip (up to 20 strokes, avoiding bubbles) and was left for 5 min at room temperature, allowing bigger pieces of tissue to settle on the bottom of the tube. The cell suspension (without bigger pieces of tissue) was used for plating onto 12 mm round glass coverslips (coated with poly-l-ornithine 50 μg/ml and laminin 10 μg/ml) in 24-well plates (∼75,000 cells/well). The medium was changed to maintenance medium (MM) with 25 μm glutamic acid, 4 h after plating. MM consisted of BrainPhys Neuronal Medium (catalog #05792, Stemcell Technologies) supplemented with Invitrogen B-27 medium (catalog #17504044, Thermo Fisher Scientific; 2% final concentration), GlutaMAX 2 mm, and gentamicin 10 μg/ml. The following day, half of the medium was changed with fresh MM. Three days after plating, half of the medium was changed with fresh MM with cytosine β-d-arabinofuranoside hydrochloride (final concentration, 1–3 μm; catalog #C6645, Sigma-Aldrich). Half of the medium was changed with fresh MM every third day, and the culture could be maintained for up to 45 d. Primary neurons were transduced by adding AAVs to the wells with neuronal cells on day *in vitro* 6–9 (DIV6–9). Neurons were imaged after DIV15, at which point they were mature enough to generate action potentials.

### Electrophysiology and voltage imaging of HEK293 cells

HEK293 cells were first washed with external solution, consisting of the following (in mm): 125 NaCl, 0.5 KCl, 1 MgCl_2_, 3 CaCl_2_, 30 glucose, and 10 HEPES, pH 7.35 adjusted with NaOH (osmolality, ∼300 mOsm/kg) and then placed into the recording chamber of an Olympus BX51WI Upright Microscope filled with 5 ml of external solution. Fluorescently labeled cells were patched under infrared differential interference contrast (IR-DIC) video microscopy. Patch pipettes (5–7 MΩ) were filled with an intracellular solution containing the following (in mm): 123 potassium gluconate, 10 HEPES, 4 MgCl_2_, 0.1 CaCl_2_, 4 ATP-Tris, 0.3 GTP-Tris, 1 EGTA, and 10 phosphocreatine di(Tris), pH 7.2 adjusted with KOH (osmolality, ∼295 mOsm/kg). Whole-cell patch-clamp recordings were done in voltage-clamp configuration, where electrical signals were amplified with Multiclamp 700B and digitized with the following two input boards: (1) Digidata Series 1400A (Molecular Devices); and (2) Neuroplex (RedShirt Imaging). Optical responses to the changes in membrane voltage were simultaneously recorded with a NeuroCCD camera (NeuroCCD-SMQ, RedShirt Imaging) connected to the microscope via a 0.67× demagnifier using a 40×, 0.8 numerical aperture water-immersion objective. Cells were held at −70 mV, and, in one set of experiments, voltage was changed from that level to −100, −40, 30, and 100 mV in a series of subsequent steps, each lasting 500 ms, followed by 500 ms of the resting voltage level (−70 mV). The optical response to these voltage changes were sampled at 20 Hz. Amplitudes of optical signals (fluorescence intensities in Δ*F*/*F*, where *F* represents baseline fluorescence before application of the voltage pulse and Δ*F* represents the intensity change from the baseline fluorescence *F*) were used for evaluation of the voltage sensitivity of the indicator. In the other set of experiments, the same voltage step (from −70 to 30 mV) was held for different durations (1, 2, 4, 6, 8, 10, 20, 100, and 500 ms) to compare the speed of each voltage indicator. Optical responses to these voltage protocols were sampled at 500 and 2000 Hz. In the third set of experiments, we tried to evaluate the optical response to action potentials. Since action potentials cannot be evoked in HEK293 cells, we used already recorded action potentials (in current-clamp mode) from cortical pyramidal neurons evoked by current injection, as voltage command (Vcmd) signal (playback action potential) for HEK293 cells. Optical responses of voltage indicators were sampled at 500 Hz in this case.

### Electrophysiology and voltage imaging of cultured neurons

Primary neurons were patched with the same equipment used for HEK293 cells, with the same intracellular solution, but with a slightly modified external solution that consisted of the following (in mm): 140 NaCl, 4.2 KCl, 1.1 CaCl_2_, 1 MgCl_2_, 10 glucose, 10 HEPES, and 2 sodium pyruvate adjusted to pH 7.35 with NaOH (osmolality, ∼320 mOsm/kg). Neurons were kept in current clamp and stimulated via patch electrode to produce three action potentials separated by 100 ms. Optical responses were sampled at 500 Hz, and each sweep was repeated five times to provide traces for temporal averaging.

### Optical filters for voltage imaging

ArcLight, Bongwoori, ASAP1-3, and chi-VSFP were excited using a 470 nm LED (pE, CoolLED); an excitation filter of 480/40 nm, a dichroic filter of 510 nm, and an emission filter of 535/50 nm. In HEK293 cells, Archon-1 and BeRST1 were excited using a 637 nm laser (140 mW; Obis LX, COHERENT); no excitation filter, a dichroic filter of 640 nm, and a long pass emission filter of 664 nm. FlicR1 was excited using a broad GYR (568 ± 60 nm) LED (pE, CoolLED); an excitation filter of 510/80 nm, a dichroic filter of 570 nm, and a long pass emission filter of 610 nm.

### Intracerebroventricular injections

AAVs containing the sequence of GEVIs of interest were mixed with Trypan Blue solution, and loaded into a Hamilton syringe, attached to a mechanical micromanipulator (catalog #NMN-21, Narishige). Newborn (P0.5) mice of either sex were cold anesthetized by placing them on ice for a couple of minutes and then were positioned on the pad below the Hamilton syringe, so that the needle touches the skull surface at a location ∼0.25 mm lateral to the sagittal suture and 0.50–0.75 mm rostral to the neonatal coronary suture. The needle was then carefully inserted into the skull 2–3 mm deep via a micromanipulator. A volume of 1–2 μl of solution was slowly injected (for ∼30 s with several 3–5 s pauses) into the lateral ventricle. After the injections, bright white light was shone through the skull to reveal Trypan Blue-filled ventricles, and mice were placed on a heated pad to recover before returning them to the breeding cage. Intracerebroventricular injections using AAV vectors carrying the following constructs were attempted (*n* indicates the number of animals injected on P0.5 and then killed at P21–P80), as follows: Bongwoori-Pos6, *n* = 12 animals; ArcLightD, *n* = 16; Archon1, *n* = 9; SomArchon1, *n* = 6; ASAP1, *n* = 6; and ASAP3b, *n* = 4. Upon the mice being killed and brain slices harvested, we found no expression of SomArchon1 (*n* = 6), ASAP1 (*n* = 6), and ASAP3b (*n* = 4). FlicR1 was never obtained in an AAV vector and never injected during this project.

### Brain slice, electrophysiology, and voltage imaging

Ventricularly injected and/or transgenic mice (P21–P80) were anesthetized with isoflurane inhalation and decapitated, and brains were extracted with the head immersed in ice-cold CSF (ACSF). ACSF contained the following (in mm): 125 NaCl, 26 NaHCO_3_, 10 glucose, 2.3 KCl, 1.26 KH2PO_4_, 2 CaCl_2_, and 1 MgSO_4_. Coronal slices (300 μm) were cut from the frontoparietal cortex, and incubated at 37°C for 30 min and then at room temperature before experimental recordings. The selection criteria for brain slices used in recordings are as follows: in both transgenic animals and ventricularly injected animals, the GEVI expression was not uniform. Some cortical areas showed stronger expression than others. From each animal, we selected brain slices with the strongest expression of the voltage indicator. In injected animals, the brain slice yield per animal was low (typically 0–4 useful slices per animal). The greatest yield of fluorescent brain slices per animal (typically 6–10 slices) was obtained in transgenic animals. However, in transgenic animals, cortical layers L2/3 and L5 expressed more strongly than cortical layers L1 and L4. All optical recordings in the current project were attained in cortical layer 2/3.

In brain slices, all experimental measurements were performed at 32–34°C. Acute brain slices were transferred to an Olympus BX51WI Upright Microscope or a Zeiss Axioskop 2F microscope, and were perfused with aerated (5% CO_2_/95% O_2_) ACSF at 32–34°C. The synaptic stimulation was achieved by a computer-controlled stimulus isolation unit, IsoFlex (A.M.P.I.). The stimulation electrodes were pulled from borosilicate glass filament (outer diameter, 1.5 mm; inner diameter, 0.8 mm; resistance, ∼7 MΩ) filled with ACSF and positioned in cortical layer 2/3. Triplets of synaptic shocks at 8.3 Hz, and at 83 Hz were delivered in the same optical recording sweep, separated by a 1 s interval. The duration of a typical optical sweep was 3 s (shutter open time = 3 s). Optical traces were repeated every 15–20 s. For the excitation of brain slices, a metal halide lamp (Lumen 200, Prior Scientific) or LED (pE, CoolLed) was used. Optical filters used on brain slices are the same as described in the subsection Optical filters for voltage imaging, except for Archon1 we used the following filters: excitation filter, 605/30 nm; dichroic filter, 640 nm; and long-pass emission filter, 665 nm. The intensity of the excitation light was similar among all GEVIs tested on brain slices. Voltage optical signals were sampled with a NeuroCCD-SMQ camera (80 × 80 pixel configuration; RedShirt Imaging). The maximum full-frame sampling rate of the NeuroCCD-SMQ camera was 2000 Hz.

### Data analysis

Analysis of optical data from HEK293 cells was performed with custom-made scripts written in MATLAB (MathWorks). The scripts used an averaged intensity of the region of interest (ROI) that covered the cell body, corrected for the background fluorescence (near the cell body). Optical signal amplitudes (Δ*F*/*F*) were thus calculated using the formula: (Fs – *F*)/*F*, where Fs is the intensity of the optical signal, and *F* is the baseline intensity (both corrected for the background fluorescence). Optical traces were plotted in GraphPad Prism 6 (GraphPad Software), and the same software was used for fitting Boltzmann sigmoid to the voltage sensitivity of optical amplitudes. Calculation of the time constant (TAU) from optical traces was performed on the sections of the trace belonging to the 100 and 500 mV voltage steps. The traces were fitted with a double exponential in MATLAB, and the two time constants (fast and slow) were extracted from the fit.

Analysis of optical data from primary neurons and brain slices, including spatial averaging, exponential subtraction, and low-pass filtering, was conducted with the Neuroplex data acquisition and analysis software (RedShirt Imaging). Optical signal amplitudes are expressed as Δ*F*/*F*, where *F* represents the resting fluorescence intensity at the beginning of the optical trace (baseline), and Δ*F* represents the intensity change from the baseline fluorescence during the biological signal. No additional corrections of *F* were used for the cultured neurons or brain slice data. Results are presented as average values ± the SEM, unless otherwise stated. Statistical significance was set at *p* < 0.05. An unpaired *t* test or one-way ANOVA was used to test statistical significance. The ANOVA results were stated as F-ratio (df1, df2), where df1 is degrees of freedom between groups and df2 is degrees of freedom within groups, outliers included.

## Results

GEVIs were tested in the following three preparations: cultured neurons, HEK293 cells, and brain slices.

### Cultured neurons

We attempted to characterize the performance of GEVIs in neurons *in vitro*. In this experimental series, we generated 10 rounds of primary hippocampal neuron cultures (see Materials and Methods). On DIV6 through DIV9, neurons were transduced by AAV vectors carrying one of the following: (1) hSyn-Bongwoori-Pos6, (2) pCAG-ASAP1, (3) hSyn-ASAP2s or CaMK2-ASAP2s, (4) hSyn-ASAP3b, (5) hSyn-Archon1, or (6) hSyn-SomArchon1. Seven to 22 d after the AAV vector treatment, the neurons were transferred to the recording chamber and examined for florescence and health. Our experiments with cultured neurons were relatively unsuccessful, as we often found weak GEVI expression or none, whereas occasional bright neurons with strong fluorescence levels were most often dead. We obtained the expression of ASAP1, ASAP2s, Bongwoori-Pos6, and Archon1 in a very small number of healthy-looking neurons; typically <10 cells per coverslip. We were unable to obtain neuronal expression of SomArchon1 and ASAP3b (zero fluorescent + healthy cells per coverslip).

An important and infrequently addressed issue with sensor evaluations is cell selection. In the present study, the following criteria were used for selecting primary cultured neurons to be patched and recorded. First, the surface of the culture is screened in the fluorescence channel, in a video microscopy mode, using low-intensity excitation light and infrared camera at high gain (in this mode, a camera is more sensitive than the human eye). Since screening the surface of a 12 mm coverslip takes some time, the combination of low excitation intensity and infrared camera protects neurons from phototoxicity during screening. Upon finding a fluorescent neuron (in fluorescence channel), we switched to infrared video microscopy and examined the surface of the neuronal cell body and primary processes. We patched cells with smooth healthy-looking cell body and two or more primary processes. Neurons with resting membrane potential more positive than −55 mV, or action potentials <60 mV were discarded. Approximately 70% of neurons that we patched in this project were discarded on the basis of poor health. In our hands, a maximum of one good neuron per coverslip can be found, patched, and recorded electrically and optically, before the coverslip needed to be replaced. Hence, all neurons reported in this section were from different coverslips.

**Figure 1.**
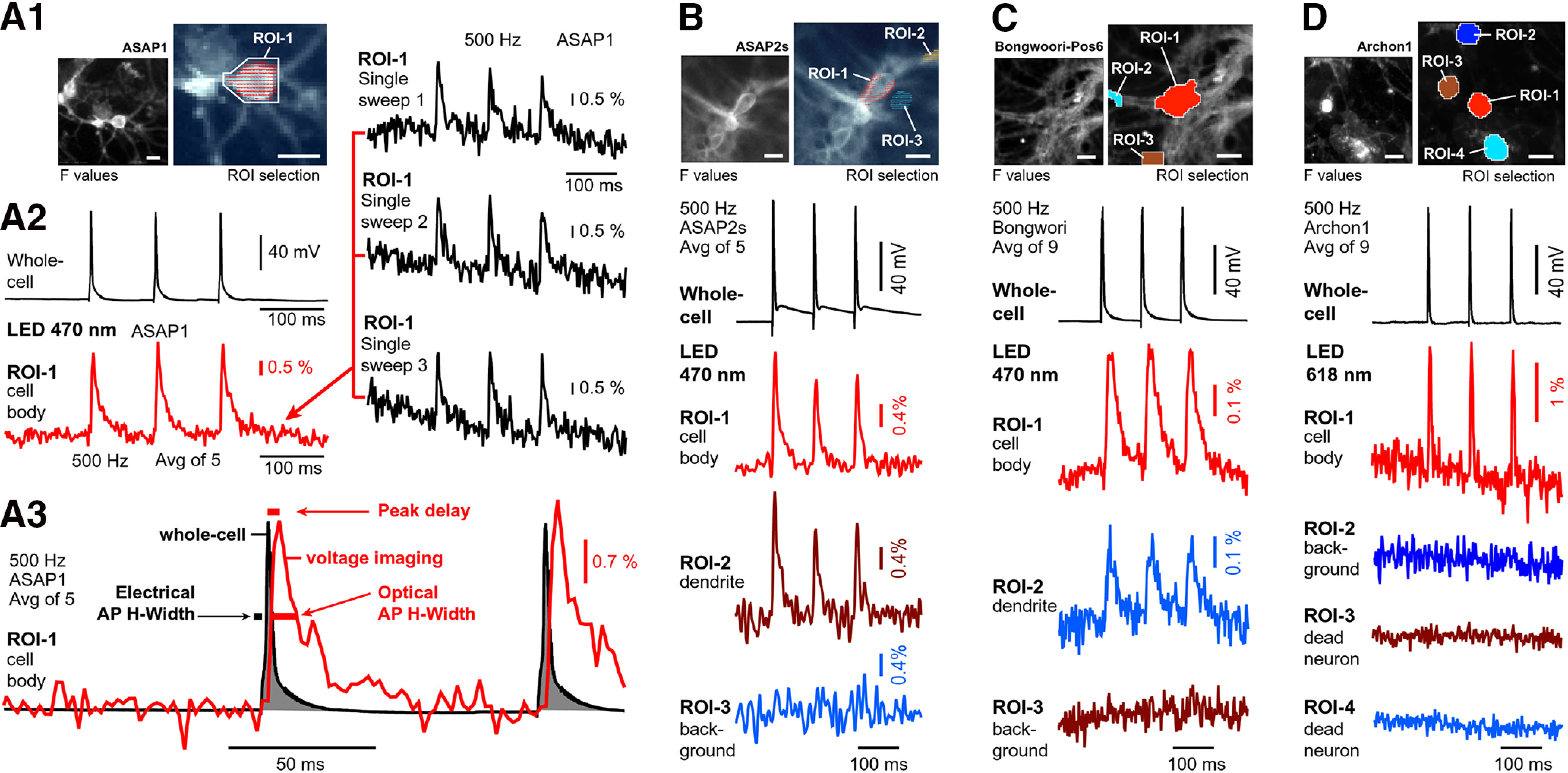
GEVI imaging in cultured neurons. ***A1***, Right, Fluorescence of primary neuron culture, mouse, DIV22, transduced with pCAG-ASAP1. Left, Image captured by a low-resolution camera during voltage imaging at 500 frames per second. ***A2***, A cell was stimulated via patch electrode to produce three action potentials, while optical signals were recorded from the entire visual field. In data display, one ROI was selected over the cell body of the patched neuron (actual pixels marked by dashes in ***A1***). Red trace shows action potential-associated optical signals after five temporal averaging, but also in single sweeps (black optical traces). ***A3***, Temporal discrepancies between electrical (gray, 16 kHz) and optical (red, 0.5 kHz) recordings: the peak of the optical signal lags behind the peak of the electrical signal (Peak delay). The action potential half-widths are much longer in optical recordings (optical action potential half-width). ***B***, Same as in ***A1–A3***, except different cell (DIV15), different AAV vector (CamK2-ASAP2s), and three ROIs are selected. ***C***, Same as in ***A–A3***, except different cell (DIV28), different AAV (hSyn-Bongwoori-Pos6), nine temporal averaging, and three ROIs are selected. ***D***, Same as in ***C***, except different cell (DIV15), different AAV (hSyn-Archon1), and four ROIs are selected. Scale bars, 10 μm. Imaging conditions for ASAP1, ASAP2, and Bongwoori: excitation filter: 480/40 nm; dichroic filter: 510 nm; and emission filter: 535/50 nm. Light power ASAP1 and ASAP2 = 5.05 mW/mm^2^; and Bongwoori = 10.1 mW/mm^2^. Imaging conditions for Archon1: LED: 568 ± 60 nm, 2.7 mW/mm^2^; excitation filter: 605/30 nm; dichroic filter: 640 nm; long-pass emission filter: 665 nm.

In live neurons expressing ASAP1, we detected action potential-associated optical signals from the cell body (Fig. 1*A1*). With illumination intensity set to 42% LED output (5.05 mW/mm^2^) and a camera sampling rate of 500 Hz, we were able to detect action potential optical signals without averaging (Fig. 1*A2*, single sweeps 1–3). At the ROI encompassing the cell body of the nerve cell, the peak of the optical signal lagged behind the electrically recorded action potential by 2.1 ± 0.2 ms (*n* = 3; Fig. 1*A3*, peak delay). The average time delay between the peak of the action potential recorded by whole-cell electrode and the peak of the same action potential recorded optically from the cell body for ASAP2s was 3.2 ± 0.1 ms (*n* = 12; Fig. 1*B*); for Bongwoori-Pos6, it was 11.2 ± 0.4 ms (*n* = 6; Fig. 1*C*); and for Archon1, it was 1.9 ± 0.2 ms (*n* = 9; Fig. 1*D*). The half-width of optically recorded action potentials was on average 6.1 ± 0.3 ms (*n* = 9), 8.5 ± 0.2 ms (*n* = 3), 12.3 ± 0.7 (*n* = 6), and 33.1 ± 2.6 (*n* = 6), respectively, for Archon1, ASAP1, ASAP2 and Bongwoori. Since we simultaneously record electrical signal via whole-cell patch electrode and optical signal via CCD camera, we compared the optical action potential half-width to the half-width of the electrically recorded action potential in the same experimental trial (Fig. 1*A3*, action potential half-width). In the case of Archon1, the relative half-width of the optical signals was on average 515 ± 51% of the electrical whole-cell signal half-width in the same trial. For ASAP1, ASAP2, and Bongwoori, this half-width distortion was on average 741 ± 25%, 766 ± 121%, and 1800 ± 122% in the optical signal. That is to say that those optical action potential waveforms were ∼5 times wider in Archon1 recordings, ∼7 times wider in ASAP1 recordings, ∼8 times wider in ASAP2s recordings, and ∼18 times wider in Bongwoori-Pos6 recordings, compared with the whole-cell action potential acquired in the same experimental trial (Fig. 1).

In summary, our data indicate that GEVIs can track electrical activity in cultured neurons on single trials (Fig. 1*A2*, single sweep). The dynamic properties of ASAP1, ASAP2s, Bongwoori-Pos6, and Archon1 impose distortions on the optical action potential timing and optical action potential duration (Fig. 1*A1–D*, ROI-1). However, the temporal distortions in the action potential timing and action potential duration (half-width) detected during the GEVI voltage imaging were negligible compared with the temporal distortions accompanying the GCaMP calcium imaging (Dana et al., 2014). Unlike BAPTA-based organic dyes such as Oregon Green BAPTA-1, GCaMP must not only bind calcium, but also undergo additional rate-limiting conformational changes before entering or exiting the fluorescent state. As a result, GCaMP fluorescence signals change at a slower rate than the binding and unbinding of calcium to its calmodulin domain (Badura et al., 2014). One of the fastest GCaMP variants, GCaMP6f, produces action potential-associated calcium signals with ∼250 ms half-widths (Badura et al., 2014), while ASAP1 produces action potential-associated voltage signals with 25 times shorter half-widths, on the order of ∼10 ms (Fig. 1*A3*). A good activity-tracking probe should minimize temporal filtering by not introducing unwanted delays (i.e., optical peak timing delayed in respect to the electrical event timing; Fig. 1*A3*, peak delay). Although we report GEVI-induced peak delays ranging from ∼2 to ∼11 ms, depending on a GEVI variant, in neurons with higher spike rates, GEVI voltage indicators such as ASAP1, ASAP2s, Bongwoori-Pos6, and Archon1 will more accurately track changes in firing frequencies, spike timing, and spike shape than any current GCaMP variant (Badura et al., 2014). Experiments in cultured neurons showed that once we were able to deliver GEVIs into the neuronal membrane, optical signals were obtained relatively easily (Fig. 1*A1–D*). However, because of very low transduction efficacy and variable expression levels in our hands, cultured neurons are not an ideal preparation for systematic testing of GEVI performance.

### HEK293 cells

**Figure 2.**
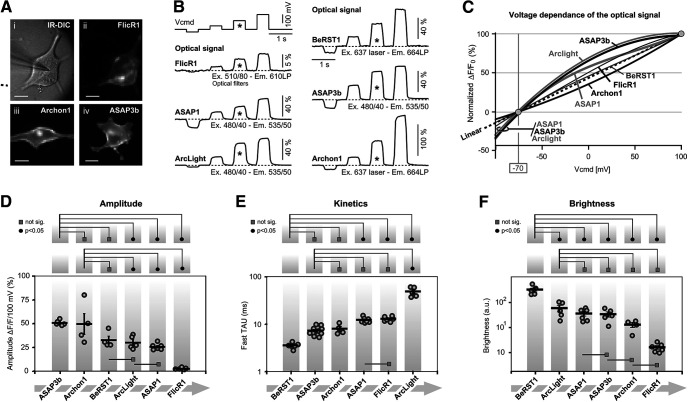
Optical signal amplitude, voltage sensitivity, and kinetics. ***A***, Microphotographs of HEK cells used for characterization of the GEVIs. ***i***, Infrared image of a cell transfected with FlickR1, with patch pipette attached. ***ii***, FlicR1 fluorescence captured by a fast (low-resolution) camera. ***iii***, ***iv***, same as ***ii***, except Archon1 or ASAP3b plasmids were used. ***B***, Each cell was voltage clamped at four command potentials (Vcmd). An asterisk marks a 100-mV-large voltage transient (from −70 to +30), which was used for reporting Δ*F*/*F* in ***D*** and Fast TAU in ***E***. Each optical trace is a product of four temporal averaging from the same cell. The best cell is displayed. Light power is reported in mW/mm^2^: FlicR1 = 1.4; ASAP1 = 2.0; ArcLight = 1.3; BeRST1 = 0.43; ASAP3b = 2.0; and Archon1 = 4.69. ***C***, Voltage sensitivity trends of six voltage indicators are superimposed. Trends are polynomial fits of the third order through the mean value of each voltage step. Each mean value is an average of four to six cells. Marker points and error bars are omitted for clarity. ***D***, Graph within borders: amplitudes of the optical signals in response to a standard 100-mV-large change in membrane potential. Each circle marker represents averaged data from one HEK cell. Thick horizontal dash is the group mean ± SEM. In this and all remaining figure panels, above the graph are displayed the results of one-way ANOVA with *post hoc* Tukey’s test. Black full circles indicate *p* < 0.05. Gray rectangles indicate no significant difference (*p* > 0.05). ***E***, Optical transient was fitted with double exponential. These Fast TAU values are plotted in the graph. Each circle marker represents averaged data from one HEK cell. ***F***, Cell body resting light intensity (basal fluorescence) in arbitrary units (a.u.).

In this experimental series, we tested the voltage sensitivity of six voltage indicators using an HEK293 cell preparation (Fig. 2*A1*). More specifically, we tested five GEVIs (FlicR1, ASAP1, ArcLight, ASAP3b, and Archon1) and one voltage-sensitive dye (BeRST1). HEK293 cells were transfected by plasmids using lipofectamine transfection protocol (0.5 μg of DNA per well of a 24-well plate) and were replated on coverslips the next day. Recordings were obtained 1–2 d after replating (Materials and Methods). We found that ASAP1, ASAP3b, and ArcLight expressed nicely, with >60% of HEK cells showing good health and strong resting fluorescence, thus yielding hundreds of good cells per coverslip. With Archon1 and FlicR1 plasmids, we found fewer GEVI-expressing HEK cells (<10 good cells per coverslip).

#### Membrane localization

All tested GEVIs exhibited a reasonably good membrane localization with a few signs of the fluorescent protein (GEVI) stuck in intracellular membranes (Fig. 2*A*,*ii*,*iii*,*iv*). We did not find a GEVI indicator with ideal membrane targeting, where 100% of protein is localized in the plasma membrane. Every GEVI in this series of experiments, showed off-target localization (e.g., intracellular membranes associated with putative Golgi lysosomes). The undesired labeling of intracellular membranes will reduce the optical sensitivity of the probe and worsen the signal-to-noise ratio in optical recordings (Popovic et al., 2015; Antic et al., 2016).

**Figure 3.**
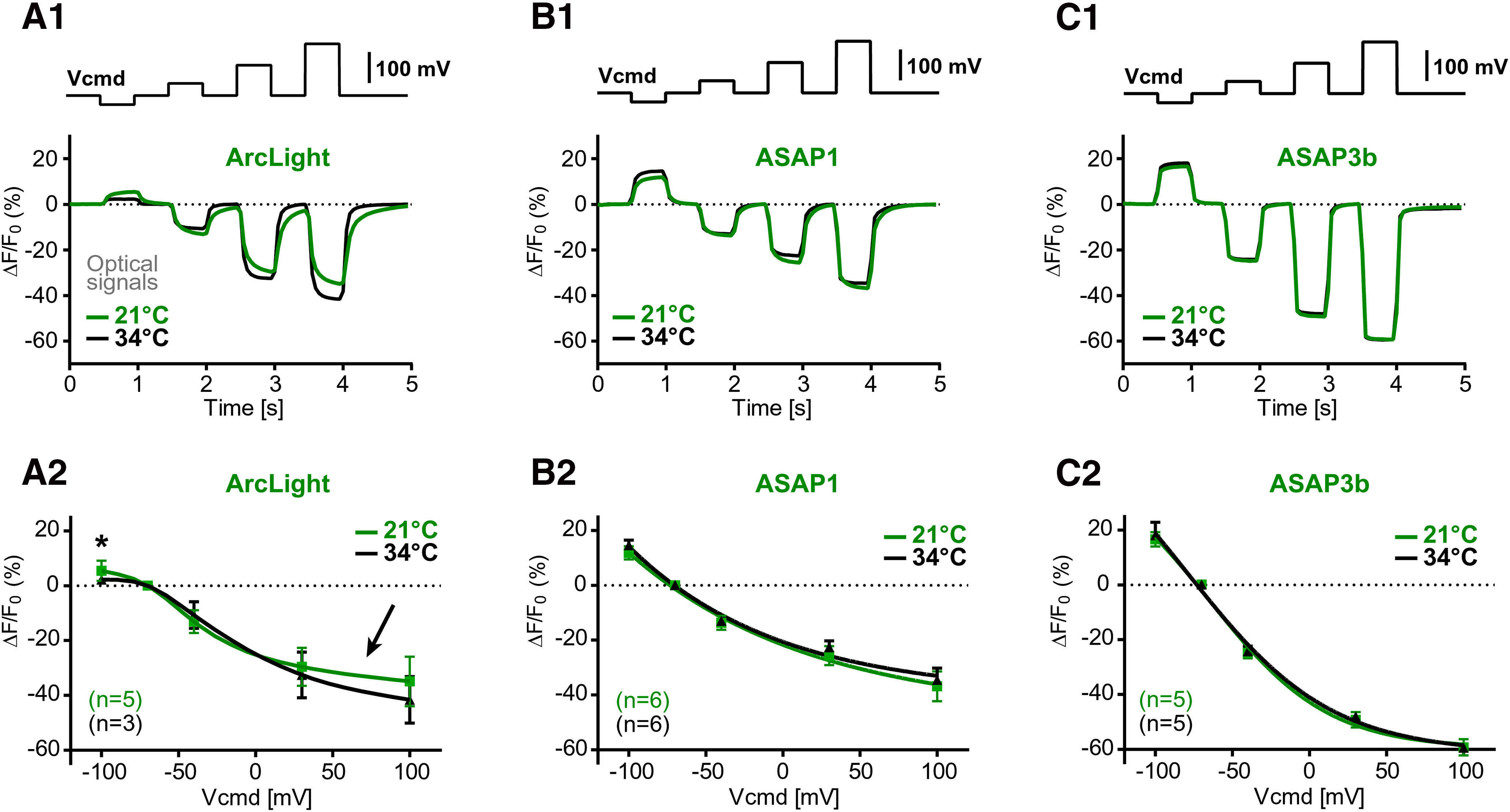
Temperature sensitivity of the GEVI optical signal. ***A1***, ***A2***, In the same cell, voltage-clamp evoked optical signals were measured at two temperatures (21°C and 34°C). Group data show voltage sensitivity of the optical signal. Each marker is a mean ± SEM. The *n* value is indicated in the bottom left corner. The trend line is a Boltzmann fit through the markers. An arrow marks the alleged discrepancy between two temperatures. An asterisk marks significant difference (*p* < 0.05). ***B1*, *B2***, Same as in ***A1*** and ***A2***, except ASAP1 was tested. ***C1***, ***C2***, Same as in ***A1*** and ***A2***, except ASAP3b was tested.

#### Voltage sensitivity

HEK293 cells transfected by GEVI variants or stained by a voltage-sensitive dye (BeRST1) were patched with 6–7 MΩ pipettes (Fig. 2*Ai*), and their membrane voltage was clamped using negative and positive command voltage steps (−30, +30, +100, and +170 mV) relative to the holding voltage, set to −70 mV (Fig. 2*B*, Vcmd). The best trace from each indicator is shown on the same amplitude and time scale, except for FlicR1 and Archon1 traces, where the amplitude scales were adjusted to fit Figure 2*B*. Optical signals from all GEVIs exhibited a more or less linear dependence on the command voltage (Fig. 2*C*). ArcLight and ASAP3b voltage dependences showed the strongest deviation from linearity (Fig. 2*C*, thick gray dashed line), consistent with the previously reported sigmoidal fluorescence–voltage relationship of ArcLight (Jin et al., 2012). Interestingly, ArcLight and two ASAP variants also gave proportionally the strongest optical signal at voltages more negative than −70 mV (neuronal resting membrane potential), suggesting that these three indicators may be useful for tracking hyperpolarizing (inhibitory) signals, consistent with the data obtained with ArcLight-derived Bongwoori (Nakajima and Baker, 2018) or ASAP1 (St-Pierre et al., 2014). The amplitudes of the optical signals obtained on a long-duration (500 ms) voltage step of 100 mV (expressed as Δ*F*/*F*/100 mV) are shown for each of the six voltage indicators, from all cells tested, on the same graph (Fig. 2*D*, gray round markers). We recorded four to nine optical traces per HEK cell. Each marker represents averaged optical data from one HEK cell. The thick black horizontal dashes denote the mean value across cells labeled with the same GEVI (group mean), and these Δ*F*/*F* values were as follows: ASAP3b: 49.3 ± 2.8%, *n* = 5; Archon1: 48.6 ± 22.4%, *n* = 4; BeRST1: 31.8 ± 8.6%, *n* = 4; ArcLight: 29.6 ± 6.9%, *n* = 5; ASAP1: 25.7 ± 3.4%, *n* = 6; and FlicR1: 2.54 ± 0.9%, *n* = 6 (Fig. 2*D*). One-way ANOVA showed that the difference between averages of some groups is statistically significant (*F*_(5,24)_ = 18.44, *p* = 1.59 × 10^−7^). In the top part of Figure 2*D*, we display the *post hoc* Tukey testing between ASAP3b and other indicators in this experimental series. ASAP3b showed significantly larger optical amplitudes than ArcLight, ASAP1, or FlicR1 (*p* < 0.05), but no statistically significant amplitude difference existed between ASAP3b and Archon1 or between ASAP3b and BeRST1 (Fig. 2*D*). In the middle row of Figure 2*D*, we display Tukey testing between Archon1 and other indicators in the experimental series. Archon1 amplitudes were not significantly different from BeRST1, but were significantly greater than the amplitudes of ArcLight, ASAP1, or FlicR1. In the base of the graph (Fig. 2*D*), we mark additional comparisons between indicators. Specifically, BeRST1 amplitudes were no different from ArcLight, and ArcLight amplitudes were no different from ASAP1 (Fig. 2*D*, gray rectangle). Overall, these data indicate that almost all tested GEVIs exhibited strong voltage sensitivity within the biologically plausible range of −100 to +100 mV (Fig. 2*C*,*D*). The apparent weak amplitude performance of FlicR1 may not be real, as discussed in the Brightness section below.

#### Kinetics of the optical response

The ON rate (Fast TAU) of the GEVI optical signal was quantified in traces in which HEK293 cells responded to a sudden voltage command step of 100 mV, and lasted ≥100 ms (Fig. 2*B*, asterisk). The probe dynamics were fitted with a double exponential equation (Materials and Methods). In Figure 2*E*, we display all recorded Fast TAU values (gray round markers) grouped per GEVI (vertical gray stripe). Each marker represents Fast TAU data from one HEK cell. The thick black horizontal dashes denote the mean Fast TAU value per GEVI (Fig. 2*E*), and these mean values were as follows, arranged from the fastest to the slowest construct: BeRST1: 3.8 ± 0.2 ms, *n* = 6; ASAP3b: 7.6 ± 0.4 ms, *n* = 12; Archon1: 8.1 ± 0.9 ms, *n* = 4; ASAP1: 11.5 ± 0.5 ms, *n* = 7; FlicR1: 11.8 ± 0.5 ms, *n* = 6; and ArcLight: 47.3 ± 4.43 ms, *n* = 5. Optical measurements shown in Figure 2*E* were performed at room temperature, using a 500 Hz optical sampling rate, which limits our temporal resolution to the Nyquist resolution of 4 ms. Consequently, all of the Fast TAU values reported here are somewhat slower than the originally reported values in the initial publications, the ArcLight response in particular. Nevertheless, our measurements and quantifications were done in a systematic manner—each GEVI variant was analyzed under identical experimental conditions. One-way ANOVA showed that the difference between the averages of some groups is big enough to be statistically significant (*F*_(5,34)_ = 104.48, *p* = 2.22 × 10^−16^). In the top panel of Figure 2*E*, we display Tukey testing between BeRST1 and other individual GEVIs used in this experimental series. The Tukey test detected a statistically significant difference between BeRST1 and ASAP1, BeRST1 and FlickR1, or BeRST1 and ArcLight (Fig. 2*E*, top, full black circle), but not between BeRST1 and ASAP3b or between BeRST1 and Archon1 (Fig. 2*E*, top gray rectangle). In the middle row of Figure 2*E*, we display Tukey testing between ASAP3b and other GEVIs in the Fast TAU experimental series. The ASAP3b kinetics was significantly faster than the kinetics of ArcLight (Fig. 2*E*, middle, full black circle), but not against any other GEVI (Fig. 2*E*, middle, gray rectangles). Both ASAP1 and FlicR1 were significantly faster than ArcLight (data not shown), while no difference was detected between ASAP1 and FlicR1 (Fig. 2*E*, middle, gray rectangle shown in the lower part of the graph). Our measurements thus indicate the following hierarchical order of speed of the indicator: BeRST1 = ASAP3b = Archon1 > ASAP1 = FlicR1 > ArcLight (Fig. 2*E*). If we instead of ANOVA with Tukey’s test, used an unpaired *t* test (data not shown), then these measurements indicate the following hierarchical order of the speed of the indicator: BeRST1 > ASAP3b = Archon1 > ASAP1 = FlicR1 > Arclight.

#### Brightness

Transfected HEK293 cells were illuminated by LED or laser (Materials and Methods). In an ROI encompassing the entire cell body, the resting light intensity (*F*) obtained before the onset of the optical signal, and adjusted for illumination intensity and camera gain, was used as a measure of the indicator brightness. In Figure 2*F*, we display all recorded brightness values (gray round markers) grouped per GEVI (vertical gray stripe). Each marker represents data from one HEK cell. The thick black horizontal dashes denote the mean brightness value per indicator, group mean (Fig. 2*F*), and these values were as follows, arranged from the most bright to the most dim (in arbitrary units × 1000): BeRST1: 329 ± 61, *n* = 5; ArcLight: 59 ± 17, *n* = 5; ASAP1: 36 ± 7, *n* = 6; ASAP3b: 34 ± 8, *n* = 5; Archon1: 13 ± 3, *n* = 4; and FlicR1: 1.6 ± 0.3, *n* = 6. One-way ANOVA detected statistically significant differences between the groups (*F*_(5,25)_ = 23.18, *p* = 1.2 × 10^−8^). In the top of Figure 2*F*, we display Tukey testing between BeRST1 and other individual indicators used in this experimental series. The brightness of voltage-sensitive dye BeRST1 outperforms all GEVIs; significant differences were detected (Fig. 2*F*, full black circles). In the middle row of Figure 2*F*, we display Tukey testing between ArcLight and other individual GEVIs. The brightness of ArcLight was not significantly different from any other GEVI (Fig. 2*F*, gray rectangles).

Next, the BeRST1 data were removed and a second ANOVA analysis was performed, this time comparing only the GEVI variants (ArcLight, ASAP1, ASAP3b, Archon1, and FlicR1). The difference between averages was statistically significant (*F*_(5,21)_ = 6.08, *p* = 0.0021). More specifically, ArcLight was significantly brighter than either Archon1 or FlicR1 (data not shown), but no difference was detected between the brightness of ArcLight against the brightness of ASAP1, or ASAP3b (data not shown).

Several factors contributed to the reported indicator brightness score including the following: inherent optical properties of the molecule, membrane expression level, excitation optical filters, emission filter, and spectral sensitivity of the optical detector. In practical terms, differences in the membrane expression level are real and contribute to the brightness character of the probe in our hands, which in turn will determine the signal-to-noise ratio in optical recordings, discussed in the studies by Lin and Schnitzer (2016) and Kannan et al. (2019); (Lin and Schnitzer, 2016; Kannan et al., 2019). All HEK293 cells were transfected in the same way, using currently available plasmids, so the comparisons shown in Figure 2*F* are informative. In our hands, the voltage-sensitive dye BeRST1 was several fold brighter than the brightest GEVI, ArcLight. The low brightness levels of FlicR1 in our current experiments suggested some practical problems with the FlicR1 plasmid, or optical filters used, and thus the FlicR1 data reporting the signal amplitude (Fig. 2*D*) and signal brightness (Fig. 2*F*) should be taken with reservation.

#### Temperature sensitivity

Some GEVI indicators use voltage-sensing domains of the naturally occurring membrane proteins (Knöpfel and Song, 2019). Channel opening and closing is a temperature-sensitive process (Hodgkin and Huxley, 1952; Rodríguez et al., 1998). We hypothesized that voltage sensitivity of the popular GEVIs (Fig. 2*C*) may fluctuate with the ambient temperature. To test this hypothesis, we ran an identical experimental paradigm (voltage clamp + voltage imaging) at two temperatures, 21°C or 34°C (Fig. 3*A1*). The mean Δ*F*/*F* values, fitted with the Boltzmann equation, revealed that ArcLight shows some temperature-dependent effects on the optical signal size, especially at the lower and upper ends of the voltage range examined (Fig. 3*A2*, arrow); however, only one voltage point (−100 mV) was significantly different between the two temperatures (Fig. 3*A2*, asterisk; unpaired *t* test, *p* < 0.05). Temperature-induced changes in voltage sensitivity were not detected when working with ASAP1 (Fig. 3*B*) or ASAP3b (Fig. 3*C*). That is, the Δ*F*/*F* versus V plots of ASAP1 and ASAP3b were stable despite the large ambient temperature change of 13°C (Fig. 3*C2*).

#### Short pulse test

In Figure 2*E*, we evaluated the speed of the optical response of each GEVI (Fast TAU) by fitting an exponential function on the optical transient, as traditionally done in the GEVI field (Jin et al., 2012; St-Pierre et al., 2014). Optical signals in the GEVI recordings are noisy, and the estimates of Fast TAU may vary among the cells expressing, for example, the same GEVI, based on signal-to-noise ratio, digital conditioning of signals (bleach subtraction), and low-pass filtering. Here we sought another method for testing the speed of fluorescence change, which would be independent from the signal-filtering parameters, incident noise levels, or the exponential fitting algorithms.

**Figure 4.**
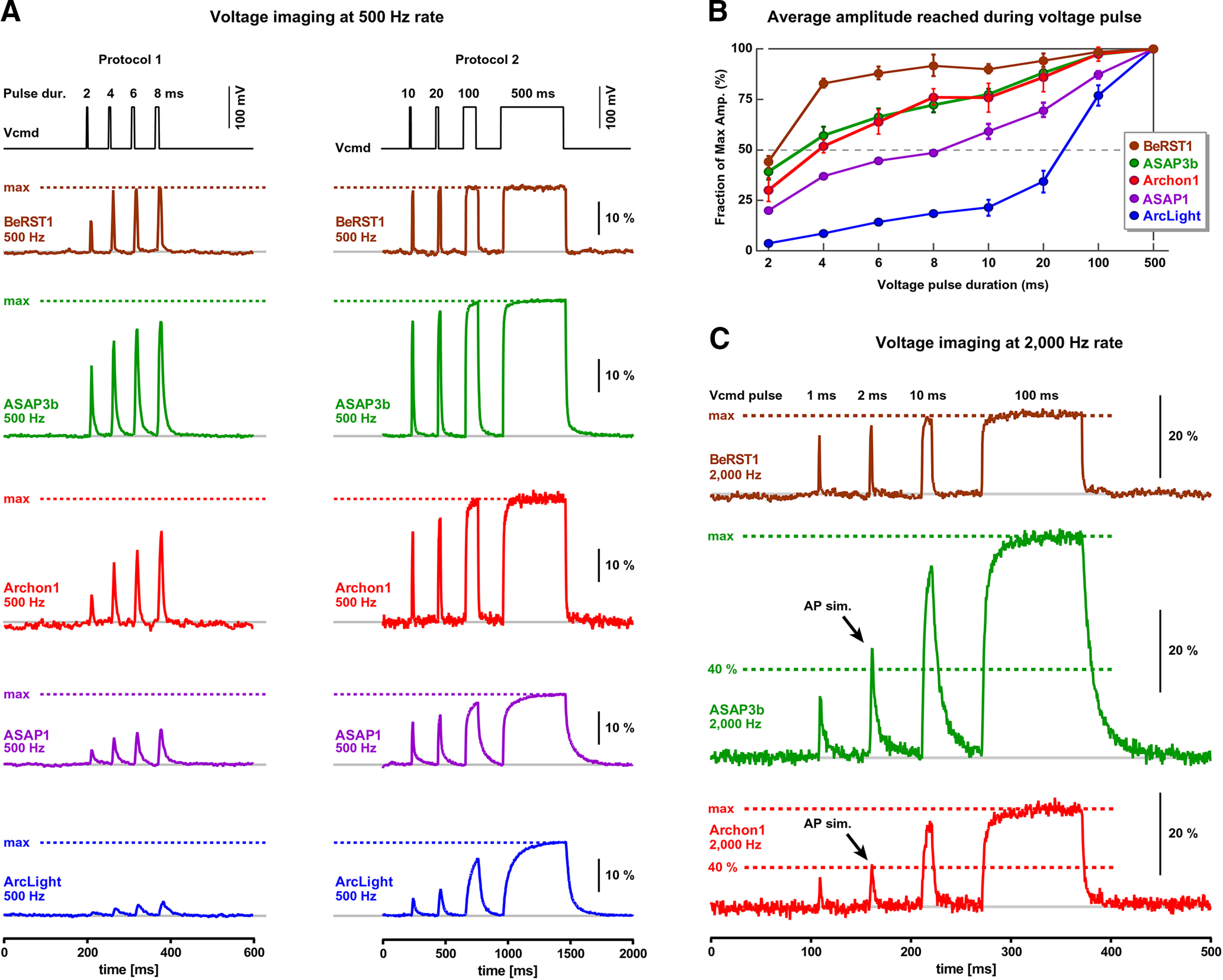
Temporal dynamics of the GEVI optical response. ***A***, Voltage pulses of the same amplitude (100 mV) but variable duration (2–8 ms, Protocol 1) were applied on HEK cells, while measuring optical signals from the surface of the cell at a 500 Hz frame rate. In Protocol 2, voltage command pulse durations were in the range 10–500 ms. All optical traces are on the same amplitude (Δ*F*/*F*) and time scale (ms). Dashed horizontal line indicates steady-state amplitude (max). ***B***, Average amplitude reached per indicator per pulse duration. Before averaging, the amplitudes were normalized using the steady-state amplitude (max) achieved during the 500 ms voltage command pulse in the same cell. The number of cells for ArcLight, ASAP1, Archon1, ASAP3b, and BeRST1 are 4, 6, 2, 6, and 3, respectively. ***C***, Evaluation of the GEVI temporal dynamics using 2000 Hz sampling of optical signals. Vcmd pulse durations are 1, 2, 10, and 100 ms. All optical traces are on the same amplitude (Δ*F*/*F*) and time (ms) scale. Steady-state amplitude (100%) and the 40% amplitude levels are marked by horizontal dashed lines to yield the interpretation of the data. AP sim. marks the optical signal responding to a 2-ms-long voltage pulse; “AP sim.” marks a 2 ms voltage pulse, which is similar in duration to a membrane potential transient experienced by the plasma membrane during a real AP.

Long (500 ms) voltage command pulses allow plenty of time for optical signals to reach their steady states, marked by the horizontal dashed lines titled “max” (Fig. 4*A*), while short (a couple of milliseconds) voltage command pulses (100 mV in amplitude) impose a considerable challenge on fluorescent indicators. That is, voltage indicators regularly failed to reach their steady-state amplitude in a short amount of time (a couple of milliseconds; Fig. 4*A*). The actual optical signal amplitude reached during a short pulse is directly proportional to the speed of the indicator (ON rate or Fast TAU). Faster indicators achieve bigger amplitudes given the same duration of time allowed (pulse duration). For the purpose of the GEVI-to-GEVI, side-by-side comparisons, we do not need absolute measurements of the Fast TAU (Fig. 2*E*). Instead, we can perform informative side-by-side comparisons using an indirect measure of the GEVI kinetics: the fraction of the steady-state amplitude reached during a standard short pulse (e.g., pulse duration = 2 ms). This finding allowed us to design a protocol for systematic testing of the GEVI kinetics in HEK293 cells without the need for exponential fitting of noisy optical traces. In the new experimental paradigm, we ordered voltage command pulses to go from short to long duration (to reduce the impact of bleaching), and we made the last pulse of this series to be long enough (e.g., 500 ms) to allow for appropriate assessment of the steady-state value (Fig. 4*A*, max). One important aspect of this experimental design is that short test pulse (2 ms) and long steady-state pulse (500 ms) are recorded under identical recording conditions (sometimes in the same trace)—to avoid, for example, variations in excitation power, excitation/emission filters, camera settings, bleaching, temperature, cell health, and voltage-clamp health. In all experiments of this experimental series, the series resistance compensation in the whole-cell recordings was adjusted to be at least 75% compensated, allowing the voltage-clamp apparatus to rapidly change the membrane voltage of HEK293 cell. To our surprise, not a single GEVI was able to reach the steady-state level even when the voltage pulse was 10 ms in duration (Fig. 4*A*, Protocol 2). This was in stark contrast to the voltage-sensitive dye BeRST1, which regularly reached ∼95% of its physical maximum (Fig. 4*A*, max) for all pulse durations, except for the 2 ms pulse. ASAP3b and Archon1 had very similar kinetic performances in our experiments, across a series of pulses, except for the 2 ms pulse (Fig. 4*B*, compare green, red), where ASAP3b responded better (faster) than Archon1. Based on the fraction of the max amplitude reached during short voltage pulses, we ranked the current voltage indicators in the following order: BeRST1 > ASAP3b > Archon1 > ASAP1 > ArcLight.

The experiments shown in Figure 4, *A* and *B*, were accomplished through voltage imaging at 500 Hz sampling rate (full frame rate). To test whether optical undersampling distorted our values shown in Figure 4*B*, we retested the three fastest indicators using voltage imaging at 2000 Hz optical sampling rate and by introducing even shorter voltage command pulses (1 ms) into the protocol (Fig. 4*C*, Vcmd pulse). The faster optical sampling rate (2000 Hz) produced identical conclusions as the slower sampling rate (500 Hz). Based on these measurements, ASAP3b and Archon1 showed the fastest responses in the group of the tested GEVIs. Assuming that the 2-ms-long voltage pulse simulates the voltage swings imposed onto the plasma membrane during a real action potential (the action potential half-width is on the order of 2 ms; Zhou et al., 2008), Archon1 and ASAP3b can capture >40% of the action potential full dynamic range (Fig. 4*C*, AP sim.). That is, during a 100-mV-tall action potential, Archon1 and ASAP3b reach at least 40% of their physical maxima for a 100 mV change, where physical maxima is the value reached during the steady state (Fig. 4*C*, max).

#### Action potential playback

**Figure 5.**
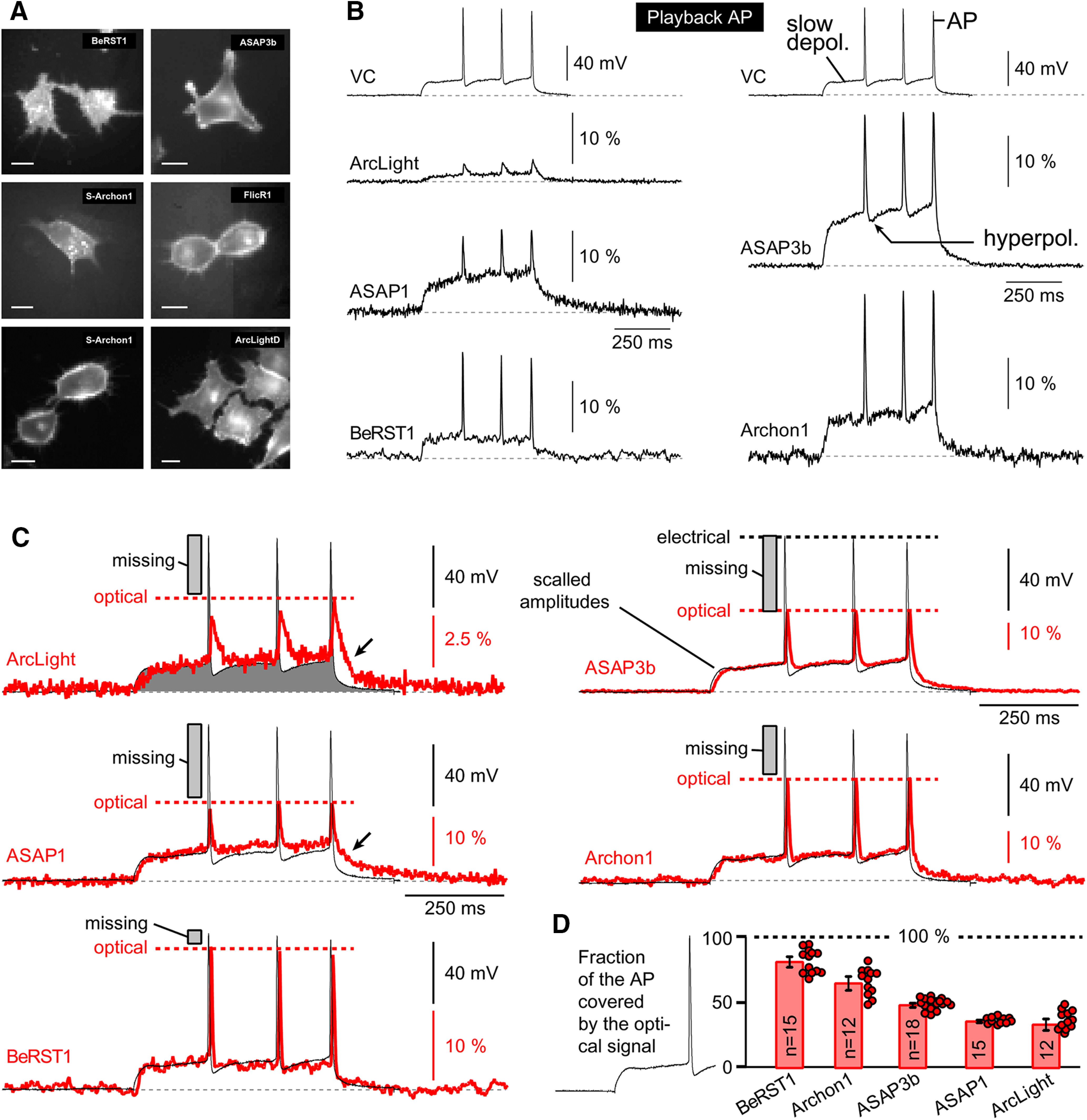
AP-Playback was used for testing the temporal dynamics of GEVIs in HEK cells. ***A***, Microphotographs of fluorescently labeled HEK cells used for testing the speed of the optical response. Scale, 10 μm. ***B***, Top, Action potential waveform recorded in cortical pyramidal neuron (in current-clamp mode) was used to shape the voltage command pulse in HEK cells (voltage-clamp mode). Bottom, Optical signals obtained from the surface of the patched HEK cell transfected with a corresponding GEVI. Each trace is a product of four temporal averaging. Optical signals were sampled at 500 Hz. All traces are shown on the same time and amplitude scale. ***C***, Slow components of electrical and optical signals are scaled. Horizontal dashed line (optical) marks the fraction of action potential covered by the GEVI optical signal. ***D***, Mean fraction of the action potential covered by the optical signal. *n* indicates the number of action potentials averaged.

We sought to develop another experimental paradigm for testing the ability of GEVIs to track an action potential, while avoiding the practical obstacles associated with the variable health of cultured neurons and the variable efficacy of GEVI transfections (Fig. 1). To this aim, we recorded action potential waveforms in cortical pyramidal neurons (Fig. 5*B*, VC), and we stored those waveforms in the computer used for patching HEK293 cells. Next, the GEVI-expressing HEK293 cells (Fig. 5*A*) were patched and voltage clamped. Instead of standard rectangular voltage steps (Fig. 2*B*), here we used prerecorded action potentials. In addition to the three fast action potentials, this trace also contained a substantial slow component (Fig. 5*B*, slow depol.). As previously noted, slow sustained depolarizations allow GEVIs plenty of time to reach the steady-state optical signal. Having both slow and fast signals in the same command voltage waveform allows for an extraction of a number of interesting features. In the same trace, we can compare the known amplitude of the slow depolarization (in millivolts) against the amplitude of the slow depolarization optical signal (in Δ*F*/*F*), but also the known amplitude of action potential (in millivolts) against the amplitude of the action potential optical signal (in Δ*F*/*F*). Furthermore, the command voltage waveform contained a natural afterhyperpolarization following the first and second action potentials (Fig. 5*B*, hyperpol.). We noted that the ability of GEVIs to track an action potential afterhyperpolarization event was determined by the OFF kinetics, but also by the signal-to-noise ratio of the optical signal. The trace with the best signal-to-noise ratio (ASAP3b) also had the best display of the afterhyperpolarizations (Fig. 5*B*, hyperpol.). The OFF kinetics of ArcLight and ASAP1 appeared the slowest in this group of indicators (Fig. 5*C*, arrows). This could explain the apparent lack of the afterhyperpolarization event in the optical traces of these two indicators (Fig. 5*B*, ArcLight, ASAP1). While Figure 5*B* depicts five voltage indicators on the exact same amplitude scale (Δ*F*/*F* in percentage), which is useful for the side-by-side optical signal comparisons, Figure 5*C* displays the optical GEVI signals scaled based on the slow depolarization phase of the optical trace. This type of data analysis revealed the fraction of the electrical action potential not covered in the optical trace (“missing”). The voltage-sensitive dye BeRST1 typically covered 81 ± 4% (*n* = 15 action potentials in five cells) of the action potential amplitude measured above the sustained plateau depolarization. The tested GEVIs Archon1, ASAP3b, ASAP1, and ArcLight covered 64% (*n* = 12 action potentials in four cells), 48% (*n* = 18 action potentials in six cells), 36% (*n* = 15 action potentials in five cells), and 33% (*n* = 12 action potentials in four cells) of the fast action potential signal, respectively (Fig. 5*D*). Based on this “AP coverage efficacy,” we ranked voltage indicators as follows: BeRST1 > Archon1 > ASAP3b > ASAP1 > ArcLight.

### Brain slices: compound synaptic potential

**Figure 6.**
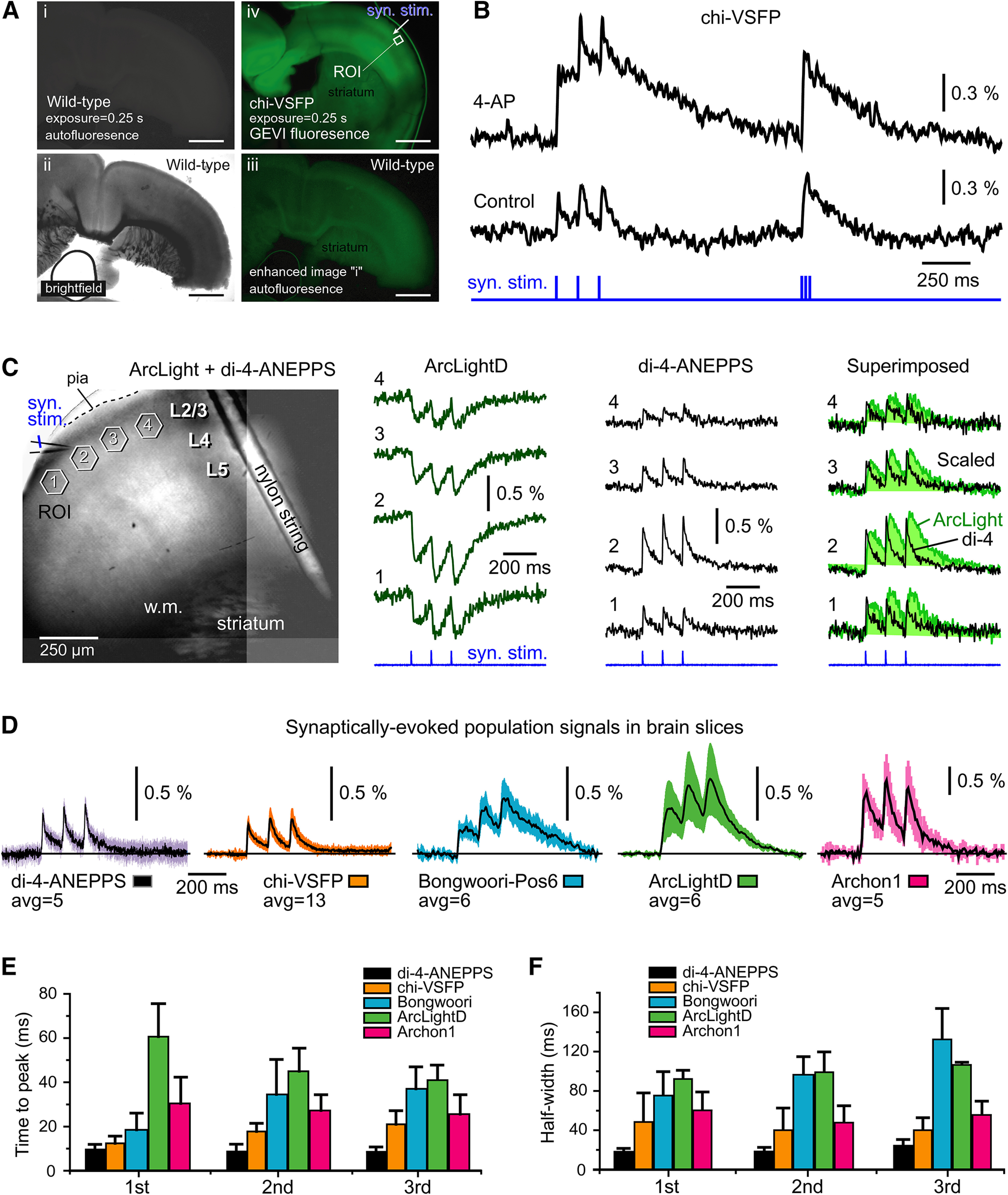
GEVI imaging of population signals in brain slices. ***A***, Coronal sections through mouse cerebral cortex. Scale bars, 1 mm. ***i***, Wild-type animal; GFP fluorescence channel; exposure duration = 0.25 s. ***ii***, Same as in ***i*** except a bright field. ***iii***, Image shown in ***i*** is enhanced digitally to reveal autofluorescence pattern in wild-type mouse cortex. ***iv***, Transgenic animal showing expression of a GEVI called chi-VSFP; GFP fluorescence channel; exposure duration = 0.25 s. Imaging settings are identical to those in ***i***. Arrow indicates the position of synaptic stimulation electrode (syn. stim.) in layer 2/3. ***B***, Synaptic stimulation was delivered in layer 2/3. Two optical traces were recorded from the same ROI. Trace Control, Before drug application; trace AP-4, after treatment with K^+^ channel blocker 4-AP (0.5 mm), the GEVI optical signal exhibits a notable increase in amplitude and duration. ***C***, Brain slice from an AAV-ArcLightD-injected animal was also stained with a voltage-sensitive dye, di-4-ANEPPS. A glass pipette used for stimulation is marked by the syn. stim. label. Optical signals were obtained from four ROIs simultaneously, first in the green channel (ArcLight channel, 480/60, 515, 535/40) and then in the red channel (di-4-ANEPPS channel, 510/60, 570, 600lp). Optical signals are products of temporal averaging (four sweeps). ***D***, Voltage indicators were delivered by the following three methods: (1) transgenic animal (chi-VSFP mouse); (2) intracerebroventricular injections of AAVs at P0 (ArcLight, Bongwoori, and Archon); and (3) incubation of brain slices with a voltage-sensitive dye di-4-ANEPPS. Regardless of the voltage indicator, each brain slice was subjected to synaptic stimulation in layer 2/3, three synaptic shocks at 120 ms interval. Optical signals are products of averaging between slices (*n* in the range 5–13). ***E***, An average time-to-peak ± SEM for each of the three synaptically evoked voltage transients. ***F***, Same as ***D***, except the quantification was made on duration (half-width) and then averaged between slices (*n* indicated in ***C***).

Acute brain slices (300 μm) prepared from the brains of transgenic animals (chi-VSFP; Fig. 6*A*,*iv*) or ventricularly injected animals (Fig. 6*C*) were positioned in the recording chamber under the 10× or 20× water-immersion objective lens. The synaptic stimulation protocol consisted of two triplets of synaptic shocks, with the interstimulus interval set to 120 and 12 ms, respectively (Fig. 6*B*, syn. stim.). In the first experimental trial (Control), synaptic stimulation, delivered in layer 2/3, evoked optical responses in the same cortical layer, 100–200 μm away from the stimulation site (Fig. 6*B*, Control). In the second experimental trial, a blocker of K^+^ channels 4-AP (0.5 mm) was introduced into the recording chamber, and voltage imaging was repeated in the same ROI and with the same synaptic stimulation protocol (Fig. 6*B*, syn. stim.). The pharmacological block of K^+^ channels with 4-AP caused a >100% increase, in both the amplitude and duration, of the first optical signal in the train (*n* = 3 animals; Fig. 6*B*, compare 4-AP, Control), consistent with an increased excitability of cortical neurons (Hoffman et al., 1997). These data indicate that GEVI imaging was not an experimental artifact, but rather the optical signal reflected an increased level of depolarization in neurons. Treatment with 4-AP may have also caused an increase in the number of synaptically recruited neurons inside the ROI, which in turn increases the amplitude and duration of the compound optical signal.

In wild-type animals, GEVIs were delivered via ventricular injection of AAVs (Materials and Methods), resulting in nonspecific labeling of the cerebral cortex—cortical neurons of all layers expressing a fluorescent indicator (Fig. 6*C*, left). Synaptic stimulations delivered in layer 2/3 produced optical signal in multiple ROIs (ROIs 1–4). In Figure 6 (unlike the previous figures), we display ArcLight optical signals in their real polarity—the ArcLight optical signal decreases with membrane depolarization. We took the negative signal polarity of ArcLight as an opportunity for combining GEVI imaging with voltage-sensitive dye imaging, in the same brain slice. In this series of experiments, following a GEVI imaging session (Fig. 6*C*, ArcLightD), the brain slices were loaded extracellularly with the voltage-sensitive dye di-4-ANEPPS (staining performed inside the recording chamber), and voltage imaging sessions were conducted using the red optical filters (Fig. 6*C*, di-4-ANEPPS). Since the X–Y position and the optical focus of the brain slice were kept fixed between green and red voltage imaging sessions, this allowed us to compare green and red optical signals in the same ROI (Fig. 6*C*, Superimposed). Optical signals obtained with green GEVIs (ArcLightD, *n* = 4; VSFP, *n* = 4; and Bongwoori, *n* = 3) invariably exhibited longer durations (duration at half-amplitude, half-widths) compared with the di-4-ANEPPS red optical signals obtained in the same brain slice, same ROI (Fig. 6*C*, Superimposed), which is consistent with the fact that organic voltage-sensitive dyes have faster response times than the protein-based GEVIs (Yan et al., 2012; Popovic et al., 2015).

Regardless of the voltage indicator used (di-4-ANEPPS, chi-VSFP-Butterfly, Bongwoori-Pos6, ArcLightD, or Archon1), each brain slice subjected to synaptic stimulation in layer 2/3 produced clear optical signals (Fig. 6*D*). Optical signals displayed in Figure 6*D* are shown on the same amplitude scale, except Archon1 where the scale is twofold reduced to fit the figure panel. Optical signals were averaged temporally (four sweeps) and then across several brain slices (*n* in the range of 5–13 brain slices). These signals are shown without low-pass filtering and on the same time scale to allow side-by-side comparisons of the temporal dynamics of GEVIs. Visual inspection of the optical traces suggested that the voltage-sensitive dye (di-4-ANEPPS) produced the fastest transients, while ArcLightD and its derivative Bongwoori-Pos6 produced the slowest responses (e.g., temporal distortion), but robust optical signals. The ArcLight signal-to-noise ratio was the largest in brain slices (Fig. 6*D*), although ArcLight amplitudes in HEK293 cells were among the smallest (Fig. 4*A*). The voltage sensitivity expressed as Δ*F*/*F* was best in Archon1 and worst in chi-VSFP experiments. More specifically, in our current brain slice assay based on synaptically evoked compound population signals, the indicator sensitivity was ranked as follows: Archon1 > ArcLightD > Bongwoori-Pos6 > di-4-ANEPPS > chi-VSFP. The acquired Δ*F*/*F* values in the current study were not strikingly different between GEVIs (Fig. 6*D*), similar to their reported differences observed in individual neurons (Chamberland et al., 2017; Xu et al., 2017). Therefore, some modifications of our experimental design and improvements in experimental success rate, involving stronger neuronal expression, improved light sources, optical filters, and optical detectors, may improve Δ*F*/*F* of selected GEVIs, and cause some reordering of the ranks.

For each of the three synaptically evoked voltage transients at 120 ms interval, we measured time-to-peak using the individual brain slice data. The fastest rise of the optical signal was detected in experiments using the voltage-sensitive dye, while the slowest rise time was detected in experiments using ArcLightD (Fig. 6*E*). Measurements of the signal duration (signal half-width) indicated that ArcLight and Bongwoori produced the broadest optical signals (Fig. 6*F*). Despite clear differences between indicators in terms of the quality of optical signal (brightness, sensitivity, speed) reported in the literature (Storace et al., 2016; Chamberland et al., 2017; Xu et al., 2017; Bando et al., 2019; Kannan et al., 2019) and evaluated in Figure 6,*E* and *F*, voltage imaging of synaptically evoked compound population depolarizations (Fig. 6*B*,*C*) is quite doable with all four genetically encoded voltage indicators tested here (Fig. 6*D*).

## Discussion

Compared with the family of calcium indicators GCaMPx, voltage indicators (GEVIs) produce weak optical signals, and, for that reason, they have, so far, yielded very few relevant systems neuroscience data. In theory, the potential of GEVIs (for neural circuit analysis) is very high (Antic et al., 2016; Knöpfel and Song, 2019). For this reason, a lot of effort has been invested in improving the performances of the existing GEVI variants, as well as in developing new ones (Lin and Schnitzer, 2016; Storace et al., 2016; Xu et al., 2017; Kannan et al., 2019). While the transfer of the GCaMP-based methods between laboratories appears to be flawless and leading to a constant stream of high-value biological data (Bai et al., 2017; Liang et al., 2018; Kerlin et al., 2019), the propagation of GEVIs between laboratories is less efficient. For this reason, it is useful to know how different GEVIs perform in the hands of a third party (Inagaki and Nagai, 2016; Lin and Schnitzer, 2016; Chamberland et al., 2017; Bando et al., 2019).

### Testing GEVIs in neuron culture

Experiments in cultured hippocampal neurons revealed our lack of laboratory skills in obtaining robust expression of GEVIs by transduction of neurons with AAV vectors. When neuron transfection occasionally did work, we obtained voltage optical signals of reasonable quality (Fig. 1), suggesting that in certain experimental designs (improved AAV transduction, or neurons isolated from transgenic GEVI animals), both evoked (Hochbaum et al., 2014) and spontaneous (Jin et al., 2012; St-Pierre et al., 2014) electrical activity of cultured neurons can be monitored by a multicell GEVI imaging technique—recordings from multiple cells simultaneously. However, for the purpose of the systematic evaluations of the biophysical characteristics of the GEVI, this particular biological preparation (neurons in culture) is not an ideal preparation. The neuron cultures are more variable than HEK293 cultures (variable cell maturity and health); the success of transfections and transductions is relatively low (low experimental yield); the transfected neurons express variable levels of a GEVI indicator in their membrane (variable brightness and signal-to-noise ratio); and in many cases, neurons expressing high levels of GEVI in their plasma membrane were injured or dead.

### Testing GEVIs in HEK293 cells

We expressed five GEVI variants in HEK293 cells and performed optical imaging of the voltage waveforms applied through a whole-cell patch pipette (Fig. 2). All of the results in the current study were obtained on the same electrophysiology imaging station centered on an upright microscope with a 40× objective and CCD camera. Compared with neuron cultures, the HEK293 cells show less variability in cell health and less variability in the expression of GEVIs in their plasma membrane (Fig. 2*F*). The ease of HEK293 transfection, and the efficacy of optical imaging in cultured cells, render this preparation well suited for testing new GEVI variants (Akemann et al., 2001; Jin et al., 2012; St-Pierre et al., 2014; Lee et al., 2017). On the positive side, all tested GEVI variants performed well in our hands, and their optical signals were in the range previously reported by the laboratory of origin. In terms of signal amplitude (Δ*F*/*F*/100 mV), the best signals in our hands were from the voltage-sensitive dye (BeRST1) and two GEVIs (ASAP3b and Archon1). In Figure 2*B*, we show the best traces. In Figure 2*D*, we show the average response (thick horizontal dash). We feel that both displays (best trace and average) are useful for evaluating the full potential of a voltage probe. Interestingly, two of the three best performing voltage indicators in our hands (Archon1 and BeRST1) were red-shifted indicators, excited by a red laser. In experiments on HEK293 cells, the 637 nm red laser (Fig. 2, Archon1) delivered approximately four times stronger light power at the object plane compared with the 470 nm blue LED illuminator (Fig. 2, ASAP3b). The HEK293 cell culture background fluorescence (autofluorescence) at green emission wavelengths (ArcLight, ASAP1, ASAP3b) is stronger than the background fluorescence at the far-red emission spectrum (Archon1 and BeRST1), thus influencing the Δ*F*/*F* values. In brain slices and intact brain tissue *in vivo*, the green autofluorescence becomes notably stronger and more disruptive to optical imaging. For that reason, empowering calcium and voltage indicators with a red emission spectrum has been recognized as a promising trend in experimental neuroscience (Perron et al., 2009; Abdelfattah et al., 2016; Dana et al., 2016; Baloban et al., 2017). In summary, three factors, (1) the excellent inherent properties of Archon1 and BeRST1, (2) the strongest illumination intensity supplied, and (3) the red-shifted excitation and emission spectra, are contributing to their performance in the best trace category (Fig. 3*B*).

The Fast TAU for ArcLight in Figure 2*E* is longer than that reported in the study by Jin et al. (2012). All measurements in Figure 2 were performed at room temperature. Room temperature improves the stability of electrode patch, reduces mechanical vibrations, and increases duration of a dual electrical–optical recording session per cell. While the Fast TAU measurements (Fig. 2*E*) cannot be used as specifications of the speed of the GEVI in mammalian brain at physiological temperature, they are useful documents of indicator performance. All measurements reported here were conducted under the same conditions and may be used for relative comparisons between indicators. In the terms of the ON rate (Fast TAU), none of the GEVIs could compete against the voltage-sensitive dye BeRST1 (Fig. 2*E*). The two slowest indicators, ArcLight and ASAP1, were repeatedly the slowest on three independent tests of the optical kinetics, as follows: test 1, the long pulse test (Fig. 2*B*); test 2, the short pulse test (Fig. 4); and test 3, the action potential-Playback test (Fig. 5). The long pulse test and exponential fitting of the signal rise (Fig. 2*E*, test 1) is well established (Jin et al., 2012), but our combined data indicate that all three tests of the kinetics of the indicator (1–3) are useful for evaluation of the biophysical properties of the GEVI. The recently developed “short pulse test” (Fig. 4, test 2) is very practical. It eliminates the exponential fitting step, and, most importantly, this test is less sensitive to noise and noise filtering than the traditional (Fig. 2*E*, test 1). The recently developed “AP-Playback test” (Fig. 5, test 3) exposes three interesting features of the GEVIs. First, a well defined slow component and several fast action potential-mimicking fast components in the same sweep (Fig. 5) can be used to calibrate the amplitude of the optical signal in millivolts. Steady states of the slow (long-duration) voltage transients are often used to calibrate optical recordings (Palmer and Stuart, 2009; Holthoff et al., 2010; Popovic et al., 2014). Second, the natural action potential waveform contains a rapid repolarization phase followed by an afterhyperpolarization transient, which are useful for gauging the speed of the OFF rate of the indicator and its ability to track hyperpolarizing events. In the cerebral cortex, hyperpolarizing potentials may provide important clues about the status of outward potassium currents (which are often influenced by important neuromodulators; Hoffman and Johnston, 1998; Dong et al., 2004), as well as the status of the GABAergic inhibitory signals (which are slower and do not require a fast response (Tremblay et al., 2016)). In an elegant study by Nakajima and Baker (2018), the spreading hyperpolarization, due to GABAergic inhibition, was optically monitored in hippocampal brain slices using GEVIs (Nakajima and Baker, 2018). Along these lines, specific mutations in the voltage-sensing domain have been shown to improve the sensitivity of the GEVI to hypopolarizing potentials (Dimitrov et al., 2007; Piao et al., 2015). Third, the AP-Playback test revealed the fraction of the action potential waveform being lost in the GEVI measurements. The speed of the action potential upstroke and downstroke is simply too fast for any given GEVI to follow faithfully. As a result, the GEVI optical signal reaches a certain height (Fig. 5*C*), which is only a fraction of the signal height that could have been reached if the optical signal were as fast as the whole-cell recording.

### Testing GEVIs in brain slices

The major difficulty in this experimental series is unstable expression level of the tested GEVIs. In our hands, the ventricular injection method had a low efficacy (a high number of failures) and a variable level of the GEVI expression. The uneven efficacy of GEVI expression from experiment to experiment precluded very precise comparisons of the performances of GEVIs. However, the acquired traces and numerical analyses (Fig. 6) do provide some useful insights. For example, the brain slice data clearly showed that all voltage indicators tested in the current study can be successfully used for monitoring synaptically evoked compound depolarizations in the neocortical neuropil (Fig. 6*D*). This type of population imaging (dense cellular labeling, no cellular resolution) produces strong and slow optical signals, which impose a minimal challenge to a voltage indicator. Note that in our brain slice experiments, the amount of the indicator-labeled neuronal plasma membrane greatly exceeds the amount of the membrane available to those who use GEVIs for monitoring membrane potential changes in individual neurons (Adam et al., 2019; Piatkevich et al., 2019; Villette et al., 2019). Furthermore, a synaptically evoked compound cortical signal is slower than an action potential, which allows for longer sampling intervals, more photons collected, and thus a better signal-to-noise ratio. For all these reasons, imaging compound population signals (Fig. 6) is less challenging than imaging electrical activity in individual neurons (Fig. 1). In the context of a compound electrical signal (population imaging without cellular resolution in a densely labeled preparation; Fig. 6), some unsuspected GEVI characteristics are more important than others. For example, two factors, (1) the sensitivity (dF/*F*/100 mV) and (2) the ON rate of the voltage indicator, are less important than (3) the ease of transfection and (4) the inherent brightness of the probe. That is to say that one can produce very useful physiological data using a probe that is neither the most sensitive nor the fastest indicator available (Fig. 6). Accordingly, Bando et al. (2019) determined that although a rhodopsin-based GEVI, QuasAr2, showed the highest signal amplitude, signal-to-noise ratio, and temporal resolution *in vitro*, it was ArcLight-MT that emerged as the most useful GEVI for measuring responses to visual stimulation *in vivo* (Bando et al., 2019). Although ArcLight is neither the fastest nor the most sensitive GEVI around, Storace et al. (2019) use ArcLight for its excellent brightness and high targeting efficacy to the neuron class of their interest. In accordance to this *in vivo* performance (Bando et al., 2019; Storace et al., 2019), ArcLight had a relatively large signal-to-noise ratio in our population measurements *ex vivo*, in brain slices (Fig. 6*D*). In summary, for the purpose of imaging synaptically evoked compound potentials in the brain parenchyma (brain slices or *in vivo*), we found that any of the currently available GEVIs would do the job (Storace et al., 2016; Xu et al., 2017; Kannan et al., 2019; Knöpfel and Song, 2019) as long as that GEVI possesses reasonable brightness, and its carrier vector (AAV or transgene) is reasonably efficient in the neuronal population of interest.

## Synthesis

Reviewing Editor: Miriam Goodman, Stanford University Decisions are customarily a result of the Reviewing Editor and the peer reviewers coming together and discussing their recommendations until a consensus is reached. When revisions are invited, a fact-based synthesis statement explaining their decision and outlining what is needed to prepare a revision will be listed below. The following reviewer(s) agreed to reveal their identity: Lawrence Cohen, Yulong Li.

This is useful and likely important paper comparing the properties of geneticially-encoded voltage indicating (GEVI) proteins. Recent advances in protein engineering have generated quite a number of GEVI proteins based on distinct sensors, fluorescent protein variants, and linkers. The GEVI proteins also differ how they report a change in transmembrane voltage. Some brighten while others dim upon depolarization, still others change FRET efficiency. The authors correctly identify a gap in knowledge regarding how each GEVI protein proteins when expressed in the same system and evaluated by a common set of tests. This manuscript seeks to fill this gap by measuring sensitivity, kinetics, brightness, temperature sensitivity of several GEVI proteins in cell-based assays (HEK293 cells) and brain slices.

Your manuscript has been evaluated in a double-blind manner by two reviewers, who provided both praise and productive criticism. Below, I include the comments from both reviewers as well as a summary of the major points raised by the reviewers to guide your efforts for revision.

Major points, editor’s summary

Sample selection and biological replicates: Please indicate both inclusion and exclusion criteria for cells selected for recordings as well as brain slices selected for recordings, if practical. Please also indicate the number of HEK293 cells recorded as well as the number of coverslips/independent transfections tested for each GEVI protein. See comment from Rev1 for context.

Not all GEVI proteins tested in brain slices: As noted by Rev1, data for ASAPs, FlickR and VSFP Butterfly are not included in Figure 6 (brain slices). If practical, include data for these GEVI proteins. If not practical, provide the reader with an explanation for why these GEVI proteins were unavailable for testing or were not tested. Revise the discussion to take this gap in knowledge into account.

Rev1

Cell and preparation selection.

An important and infrequently addressed issue with sensor evaluations is cell and preparation selection. The scientists doing the screening choose which cells they are going to patch and choose which slice preparations are worth examining. The criteria used in making these choices is not always known, perhaps even to the scientist making the choice. The criteria are rarely explicitly stated. The authors did not comment about these issues. They should indicate how many cells from how many coverslips were patched and how they were chosen. Did cells or preparations with small or no signals get deleted from the data? I was suspicious about this possibility because in our laboratory there will be occasional cells/preparations with no signal with no apparent explanation and the remainder of the cells will have a considerable spread of signal size. The data shown in Figure 2D and F seem too consistent, more consistent than I would have expected from our own measurements.

There were unexpected sensor omissions from the HEK293 and the in vitro slice screens. Why are data for the ASAPs, FlickR, and VSFP butterfly not included in Figure 6? Perhaps the signals were small and the authors were afraid of antagonizing the labs which developed these sensors? Some explanation is needed. Bando et al (2019) did find that ArcLight was better than ASAP in in vivo measurements. VSFP butterfly (a FRET sensor) is mentioned in the abstract but no results are shown. Also it might be useful to test an improved butterfly, Nabi2.244 (Sung et al 2015).

The red sensors were illuminated with a light intensity four times brighter than the green sensors. This would improve the signal-to-noise ratio by a factor of 2 in a shot-noise limited measurement. The authors should correct their results in Figure 6F for this difference in incident intensity. Higher incident light intensity in in vivo measurements often results in unintended heating of an in vivo mammalian preparation.

The fast tau for ArcLight in Figure 2E is about five times longer than that reported in Jin et al. 2012 and in more recent unpublished experiments in this reviewer’s laboratory. Perhaps the room temperature values are shown? Because the real use of GEVIs in mammalian brains will be carried out at body temperature, the measurements at room temperature will only be relevant for non-mammalian preparations (or ex vivo measurements at room temperature).

Examination of the traces shown in Figure 6C shows ArcLight to have a better signal-to-noise ratio than Archon1 but the opposite is true in Figure 6F. In Figure 6C Archon1 and Bonhwoori-Pos6 have a similar signal-to-noise ratio but in in Figure 6F they differ markedly. ???

The Archon1 neurons in Figure 1D are rounded up. Is this typical? There was no comment in the text.

Many laboratories have difficulty in using acutely cultured neurons for screening GEVIs. The difficulties described here are not unexpected.

Minor comments.

• The grammar and composition of the manuscript has a number of minor flaws; Rev1 offers to proof-read the revised manuscript.

• There are too many undefined abbreviations. Abbreviations make it hard on the reader. Limit the use of abbreviations to those in very common use.

• The camera is not specified.

• In line 620, ASAP3b not 4b

• Specify the AAV injection time.

• The trace during the hyperpolarizing pulse for ArcLight in Figure 2B is missing.

•The title to Figure 2 includes “temperature sensitivity” but temperature sensitivity is not included in this figure.

• In line 593, add Jin et al., 2012 to the references.

Rev2

Overall it is a good manuscript with solid data and the info would be useful for the community and I have only minor questions. See below.

1.) In Fig. 1A3, it’s better to further enlarge the distance between the peak of AP recorded by whole-cell clamp electrode and the peak of the same AP recorded optically from the cell body for ASAP1. Under current condition, it’s difficult to see clearly the temporal discrepancies between them.

2.) In Fig. 1B, the ROIs are a little bit blurry, if possible, please change the shading color of the ROI to help readers. In addition, the shape form whole cell clamp recoding looks different from the ones in Fig. 1A, C and D.

3.) It’s better to plot the peak timing and half width of the optical signals from these four sensors in the Fig. 1, which could help the reader to see it clearly rather than words in the text.

4.) The fluorescence images in Fig. 1 is not clear enough to see the membrane trafficking of these four indicators, maybe it’s a good choice to average several images from a time lapse to show their membrane localization.

5.) In page 13 line 289 and 295, whether the n=3 is too small for in vitro cultured neuron experiments to gain reliable results.

6.) In page 15 line 340, there is a sentence “All tested GEVIs exhibited a reasonably good membrane localization with minimal signs of the fluorescent protein (GEVI) stuck in intracellular membranes (Fig. 2Aii, iii, iv)”. According to the fluorescence images of the Archon1 and FlicR1, there are obvious accumulation in organelles.

7.) In Fig. 2E, statistical test is needed between different GEVIs to support your statement of GEVIs ranking in page 17 line 383-384.

8.) In page 19 line 402, there is a sentence “The low brightness levels of FlicR1 in our current experiments suggested some practical problems with the FlicR1 plasmid, or optical filters used, and thus the FlicR1 data reporting the signal amplitude (Fig. 2D) and signal brightness (Fig. 2F) should be taken with reservation”. The plasmid info and optical filters could be double-checked to make sure they are suitable.

9.) Could you provide information of the temperature sensitivity of BeRST1, Archon1 and FlicR1, because they’re not compared with ArcLight, ASAP1 and ASAP3b in Fig.3.

10.) In the short pulse test experiment with 2000 Hz sampling rate, it’s better to make a summary just like in the Fig. 4B.

11.) In Fig. 5D, it’s a good way to use the combination of dot plot and bar graph instead of bar graph only, which could help readers to figure out the data stability.

12.) In Fig. 6A, it’s better to have a control slice without sensor expressing to compare and emphasize sensor expression regions.

13.) In Fig. 6A, is that possible to label the electrode position and recoding region just like in Fig. 6B to help the reader to understand the experiment.

14.) In page 22 line 516, ‘consistent with the an increased excitability...’ should be ‘consistent with an increased excitability’?

15.) In Fig.6F, could both individual data points and averaged bar graph be plotted to show the data stability, like figure plots in Fig. 2D, E, F.

16.) For the logic of this paper, it might be a good idea to show data of HEK 293 cells first, then cultured neurons, and finally with brain slices.
